# Application of a novel T1 retrospective quantification using internal references (T1‐REQUIRE) algorithm to derive quantitative T1 relaxation maps of the brain

**DOI:** 10.1002/ima.22768

**Published:** 2022-06-13

**Authors:** Adam Hasse, Julian Bertini, Sean Foxley, Yong Jeong, Adil Javed, Timothy J. Carroll

**Affiliations:** ^1^ Graduate Program in Medical Physics University of Chicago Chicago Illinois USA; ^2^ Department of Radiology University of Chicago Chicago Illinois; ^3^ Department of Neurology University of Chicago Chicago Illinois USA

**Keywords:** MRI, quantification, T1 relaxometry, T1‐weighted, validation

## Abstract

Most MRI sequences used clinically are qualitative or weighted. While such images provide useful information for clinicians to diagnose and monitor disease progression, they lack the ability to quantify tissue damage for more objective assessment. In this study, an algorithm referred to as the T1‐REQUIRE is presented as a proof‐of‐concept which uses nonlinear transformations to retrospectively estimate T1 relaxation times in the brain using T1‐weighted MRIs, the appropriate signal equation, and internal, healthy tissues as references. T1‐REQUIRE was applied to two T1‐weighted MR sequences, a spin‐echo and a MPRAGE, and validated with a reference standard T1 mapping algorithm in vivo. In addition, a multiscanner study was run using MPRAGE images to determine the effectiveness of T1‐REQUIRE in conforming the data from different scanners into a more uniform way of analyzing T1‐relaxation maps. The T1‐REQUIRE algorithm shows good agreement with the reference standard (Lin's concordance correlation coefficients of 0.884 for the spin‐echo and 0.838 for the MPRAGE) and with each other (Lin's concordance correlation coefficient of 0.887). The interscanner studies showed improved alignment of cumulative distribution functions after T1‐REQUIRE was performed. T1‐REQUIRE was validated with a reference standard and shown to be an effective estimate of T1 over a clinically relevant range of T1 values. In addition, T1‐REQUIRE showed excellent data conformity across different scanners, providing evidence that T1‐REQUIRE could be a useful addition to big data pipelines.

## 1 INTRODUCTION

Conventional MR images used to detect and determine the severity of disease are often qualitative in nature. The contrast in different types of tissues is enhanced or weighted arbitrarily to emphasize either anatomy or pathology of interest. Due to this arbitrary nature of signal acquisition which can vary among different MRI scanner models, it is rather challenging to compare signal intensities and their associated clinical relevance across different scanners. Even if the acquisition protocol is controlled or harmonized to the best of ability and the same scanner model is used repeatedly, voxel intensity differences may still arise from dissimilarities in coils, fields, scanner software, upgrades, and unknown effects of shielding, variables that cannot be fully controlled.

In addition to scanner harmonization, several approaches have been used to minimize variations in the unit intensities across different scanners or even within the same scanner longitudinally. A statistical approach to harmonization of downstream end results is ComBat (combating batch effects when combining batches), which uses a Bayesian method for data harmonization.^1^ Histogram matching aligns and thereby stretches or compresses the range of voxel intensities to a template.^2^ White Stripe methodology uses patches of normal appearing white matter (WM) as reference values to rescale intensities across different scanners and works well for comparing white matter hyperintensities across scanners, but it performs poorly for gray matter (GM) harmonization.^2^ Removal of Artificial Voxel Effect by Linear regression (RAVEL) methodology first uses White Stripe for intensity normalization but further decomposes the voxel intensities into a biological component and a control component such as the CSF, from which unwanted variations are assessed across images and statistically removed at the voxel level.^3^ Of note, the end result of all these intensity harmonization techniques is to reduce partial volume effects and improve volumetric analysis such as atrophy measurements.

In this study, a fundamentally different approach is taken to examine voxel intensities across different scanners. The goal is not to harmonize these voxel intensities across different scanners for downstream atrophy analysis, but to derive a quantitative measure that could potentially serve as an additional biomarker of actual tissue pathology. Rather than rescaling signal intensities linearly using various tissue references, we use a novel physics‐based approach to derive tissue relaxometry values of voxels in a nonlinear manner, which is more likely to preserve biological variability across scanners than previously used statistical methods.

Although there are many methods to producing these quantitative maps natively rather than through conversion of T1‐weighted images, they are rarely acquired clinically due to long scan times. Therefore, it would be desirable to generate estimated quantitative T1 MR maps from already acquired T1‐weighted sequences that could be easily postprocessed, and then used by clinicians and researchers as another easily obtainable marker for assessing tissue pathology. Furthermore, if this technique is validated, it could potentially lead to examining tissue pathology in previously acquired MRIs over a longer span of years than is possible in the short duration of clinical trials. T1 relaxation maps have been a useful tool for analysis of a variety of pathology, including cancer, multiple sclerosis (MS), and aneurysms.^4^, ^5^, ^6^, ^7^, ^8^, ^9^, ^10^, ^11^ A novel quantification algorithm referred to as T1 REtrospective Quantification Using Internal REferences (T1‐REQUIRE) is presented as a proof‐of‐concept that can produce estimates of quantitative T1 maps from spin‐echo T1‐weighted MR images for physics‐based data normalization. We test the hypothesis that T1‐REQUIRE will produce estimated T1 maps that are within 10% of the true T1 relaxation times over a clinically relevant range of values for neuroimaging in both a phantom study and a healthy control study.

## 2 METHODS AND MATERIALS

In this analysis, we derive a novel algorithm for retrospective calibration of individual voxels in a T1‐weighted spin‐echo MR image to true T1 values and validate using both a phantom with known T1 values and healthy controls. This was a HIPAA compliant investigation that was approved by the Institutional Review Board of our institution.

### 2.1 Theory

Signal weighted MR images produce a signal intensity based on the MRI pulse sequence (e.g., gradient‐recalled echo [GRE], spin echo [SE], echo planar image [EPI], magnetization prepared rapid gradient echo [MPRAGE], etc.) used to acquire those images. These signal equations are dependent on a variety of physical parameters (flip angle, repetition time [TR], echo time [TE], inversion time [TI], etc.) but are always dependent on the primary contrast mechanism used to achieve signal intensity differences in the image itself. For example, T1‐weighted images will always be dependent on the T1 relaxation times of the tissues in the image, otherwise it is no longer T1 weighted. Therefore, if we know the relationship between T1 and the signal intensity (either by signal equation or simulation), it should be possible to use signal intensities from reference points with known relaxation times within the weighted image to calculate the relaxation times of all voxels in that image. This should be true given that there are enough reference points to fit and that the desired relaxation time calculation is involved in the contrast mechanism of the weighted image itself. This is the basic reasoning behind the REQUIRE algorithm.

### 2.2 Algorithm design

The T1‐REQUIRE algorithm was implemented as an unsupervised image postprocessing pipeline developed in‐house using commercially available software (MATLAB v2019b, The Mathworks, Nantick, Massachusetts). The goal was to allow for the fully automated analysis of large datasets, something necessary if implementing it as a preprocessing step in a machine learning pipeline.

### 2.3 Spin‐echo implementation

To begin validating the REQUIRE algorithm, we start with the simplest sequence, a spin‐echo. Spin‐echo T1‐weighted MR images produce a theoretical signal (*S*) according to Equation (1).^12^ This means that any spin‐echo T1‐weighted image will produce a predictable signal for a given proton density *ρ*, repetition time *TR*, *T*1 relaxation time, echo time *TE*, and *T*2 relaxation time in addition to variable scanner settings *k* (transmit and receive gains, dynamic range of analog to digital [ADC] gain, etc.)

(1) S=kρ1−e−TRT1e−TET2

Because the signal is predictable for fixed settings, we can use reference literature values for *T*2 and *ρ* to artificially estimate an image (*S*_*T*1_) that is only dependent on *k* and *T*1 for a set *TR* (i.e., purely T1‐weighted). In addition, the variable scanner settings *k* should have much less variation on a voxel‐by‐voxel basis than the variation in T1 relaxation times, so the assumption that *k* is relatively constant throughout the image can be made.

(2) SeTET2ρ=ST1=k1−e−TRT1

**FIGURE 1:**
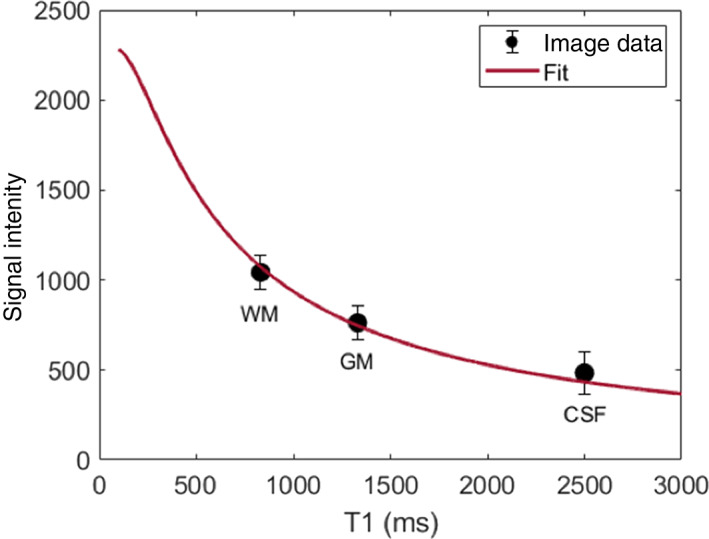
Example fit of signal intensity from corrected T1‐weighted spin‐echo (*S*_*T*1_) and relaxation times of WM, GM, and CSF

Using this knowledge, we can use the average signal intensity of each reference tissue in the corrected T1‐weighted spin‐echo image to fit Equation (2) for each image stack. Once *k* is solved for, we have a general equation that will relate any signal intensity in the corrected image to its corresponding T1 relaxation time, as shown in Figure 1.

**FIGURE 2:**
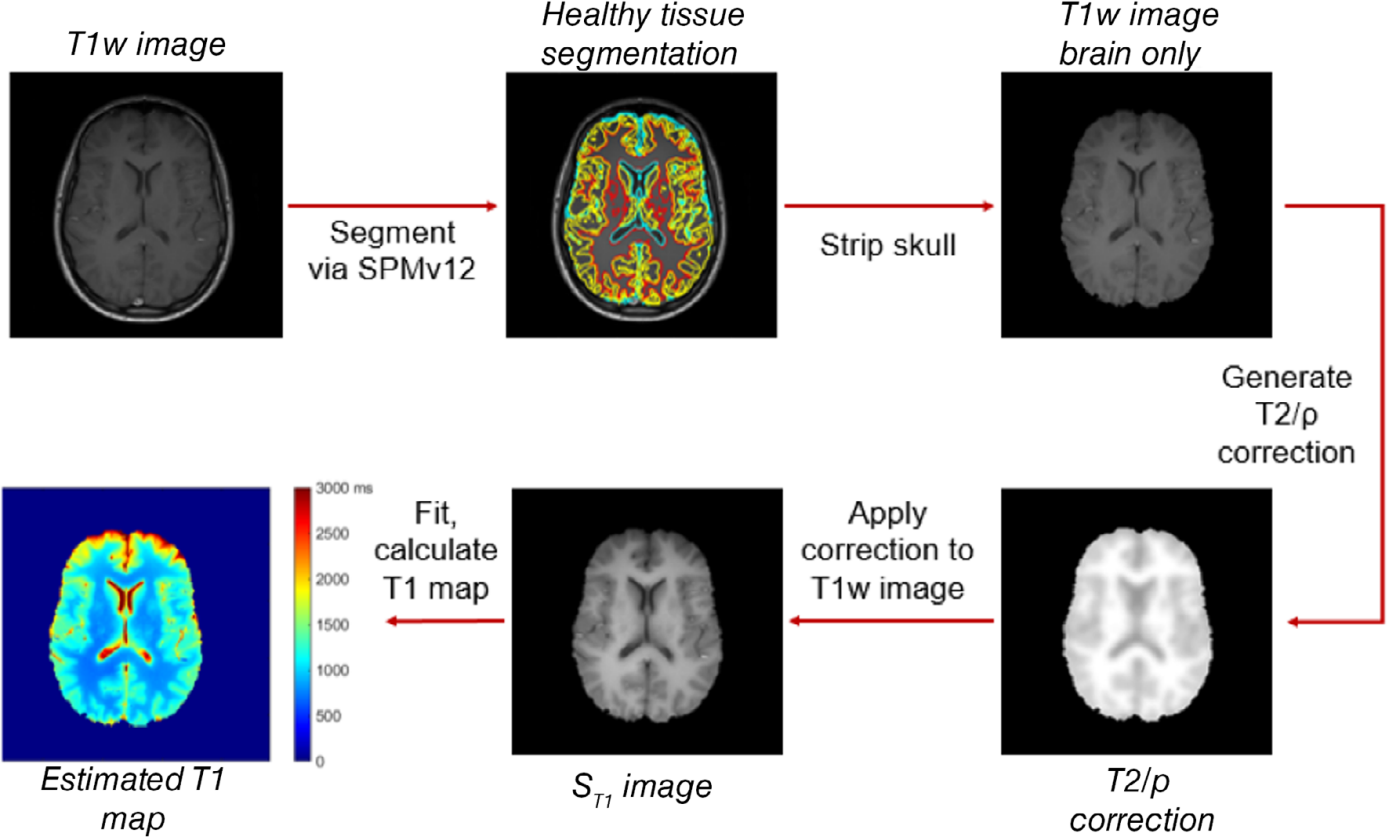
Flowchart of T1‐REQUIRE algorithm for a spin‐echo MRI

Figure 2 shows a general schematic of the algorithm design for the case of a T1‐weighted spin‐echo image. Image analysis was performed on whole brain MR images. T1‐weighted spin‐echo images were converted into NIFTI format for use with the Statistical Parameter Mapping v12 (SPM12—Wellcome Centre for Human Neuroimaging, University College London, UK) software for segmentation.^13^ Whole brain images were segmented by SPM12 into six tissue types based on anatomical and pixel intensity information. These six tissue types include gray matter (GM), white matter (WM), cerebrospinal fluid (CSF), fat, bone, and air/background. GM, WM, and CSF used as reference tissues.

Upon successful completion of image segmentation, the skull was stripped from the image by removing all tissue that was not classified as GM, WM, or CSF. A dilation and subsequent erosion were performed in addition to a hole‐filling tool in MATLAB to create a brain mask. This was then applied to the T1w spin‐echo image. To generate the dual *T*2/*ρ* correction to calculate *S*_*T*1_, voxels of each healthy tissue type were given reference literature values for *T*2 and *ρ* (Table 1).^14^, ^15^, ^16^ These were smoothed using a two‐dimensional Gaussian filter across the sagittal and coronal directions to generate the *T*2/*ρ* correction. This correction was then applied to the stripped T1w image to create the *S*_*T1*_ image necessary for fitting. The average signal intensity of each of the reference tissue type was fit to its historical T1 value (Table 1) using Equation (2) and MATLAB's nonlinear fitting algorithm to solve for *k*.^14^, ^15^, ^16^, ^17^ Once fit, the entire slice is converted to a T1 map by inverting Equation (1) and using the image signal intensities. This is done on a slice‐by‐slice basis to remove any inhomogeneities in the *B*_0_ field that alter the signal intensity along the axial direction. Because of this, only slices that have all three reference tissue types can be converted into a T1 map, a limitation overall but not for the purpose of this study.

**TABLE 1:** Healthy brain tissue MR parameters at 3 T

Tissue type	T1 relaxation time ± *σ* (ms)	T2 relaxation time ± *σ* (ms)	*ρ* ± *σ* (relative to H_2_O)
Gray matter	1331 ± 57	110 ± 8.7	0.807 ± 0.0160
White matter	832 ± 44	79.6 ± 2.6	0.679 ± 0.0168
Cerebrospinal fluid^a^	2500	1447 ± 52	1

^a^ CSF T1 relaxation time was set to 2500 to help increase contrast between GM/WM. Literature values range from 2000 to over 4000 ms. In addition, the T2 of CSF is so long that it was assumed that no T2 effects were present.

### 2.4 MPRAGE implementation

To test that the theory of the T1‐REQUIRE algorithm is applicable to any T1‐weighted image, it was tested on another, more complicated sequence: the T1‐weighted magnetization prepared rapid gradient echo (MPRAGE) sequence. Instead of a simple combination of 90–180 pulses like with a spin‐echo, MPRAGE consists of an inversion pulse followed by rapid gradient‐echo sampling to quickly produce a 3D T1‐weighted image. Because of its speed, resolution, and superior gray‐white matter contrast, it is a commonly used imaging sequence clinically.

Unlike the spin‐echo sequence, there is no simple analytical solution to invert and apply to the image. Instead, the following signal equations are used:

(3a) SN=MNe−TET2*

(3b) MN=M01−e−TRT1∑i=0N2−2cosα∙e−TRT1i+M01−e−TRT1+Meq∙eTIT1cosα∙e−TRT1N2−1

(3c) Meq=11−X∙e−TrecT1∙M01−e−TrecT1

(3d) X=1−e−TRT1∑i=0N2−2cosα∙e−TRT1i+1−2e−TIT1cosα∙e−TRT1N2−1

In Equations ((3a), (3b), (3c), (3d)), *S*_*N*_ is the signal at the *N*th phase encoding step, *M*_*N*_ is the magnetization at the *N*th phase encoding step, *α* is the flip angle, *M*_eq_ is the equilibrium magnetization, *TI* is the inversion time, *T*_rec_ is the recovery period after readout until the next inversion, and *X* is the fraction remaining as a result of small‐angle excitation.^18^ For our purposes, the signal decay due to T2* effects was ignored.

**FIGURE 3:**
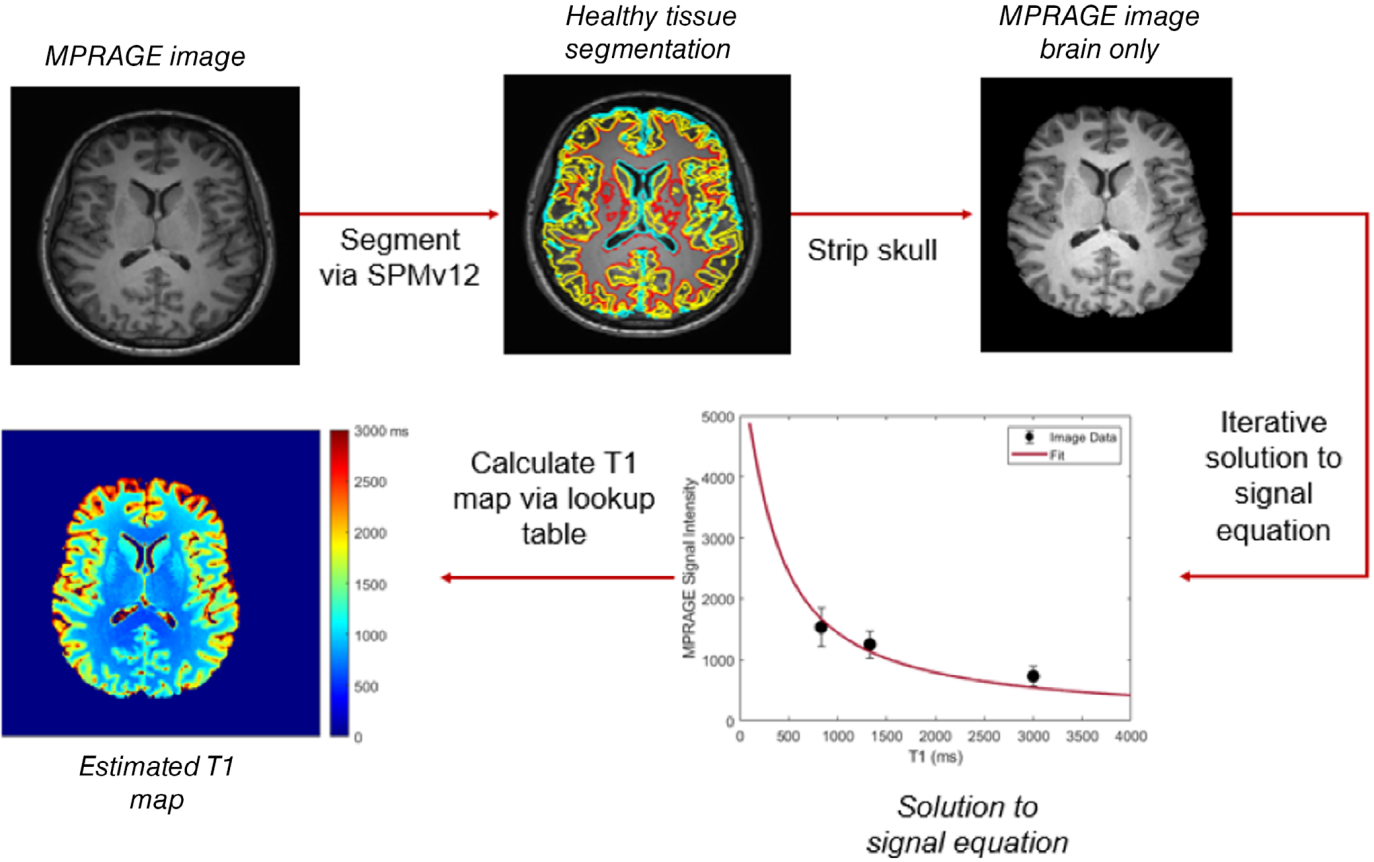
Flowchart of T1‐REQUIRE algorithm for an MPRAGE MRI

For implementation, an iterative method was used. Much like with the spin‐echo implementation, the healthy tissue types were segmented using SPMv12, with GM, WM, and CSF being used as references (Table 1). The skull was stripped and applied to the MPRAGE image. Once average healthy tissue signal intensity values were calculated, *M*_0_ was solved for using the known values for all other variables by minimizing the square error between the reference signal intensities for the healthy tissues and the calculated signal intensities. After *M*_0_ was calculated to a sufficient degree, a lookup table was generated for all T1 values and their calculated signal intensities. This lookup table was then used to generate an estimated T1 map from the MPRAGE image. Unlike the spin‐echo implementation, there is no need for all three reference tissue types to occur in every slice for this method to be applicable. A general schematic of the MPRAGE implementation of the REQUIRE algorithm is shown in Figure 3.

### 2.5 Healthy control study

Ten healthy controls (34.1 ± 10.3 years, 7 males, 3 females) were scanned on a Philips Ingenia 3 T MRI scanner at our local institution. The protocol for these studies included a T1‐mapping Look‐Locker with multiple inversion times (TE = 4.6 ms, TR = 8 ms, TI = 150, 400, 750, 1500, 3500 ms, *α* = 12°, 240 × 196 × 65 mm^3^ [240 × 240 × 13 pixels] FOV), a T1‐weighted spin‐echo (TE = 10 ms, TR = 525 ms, 240 × 196 × 65 mm^3^ [240 × 240 × 13 pixels] FOV), and an MPRAGE (*TE* = 2.89 ms, *TR* = 8 ms, *α* = 9°, *TI* = 358 ms, *T*_rec_ = 400 ms, *n* = 176, 256 × 256 × 176 mm^3^ [256 × 256 × 176 pixels] FOV). These three sequences were run back‐to‐back‐to‐back to reduce the risk of movement in between the three scans. Images were exported into MATLAB and converted into T1 maps either by the T1‐REQUIRE algorithm (T1‐weighted spin‐echo images and T1‐weighted MPRAGE images) or via Equations ((4a), (4b)).^19^

(4a) S=A−BeTIT1*

(4b) $$T1=T1^{*}\left(\frac{B}{A}-1\right)$$

### 2.6 T1‐REQUIRE with and without RAVEL harmonization of unit intensities

MPRAGE images of 10 healthy controls were processed through RAVEL prior to T1‐REQUIRE implementation to assess whether harmonization of voxel intensities would influence either the quality of T1 maps or their density distribution. T1 relaxation maps and density distribution were compared visually for the set of images processed without RAVEL and after RAVEL harmonization.

### 2.7 Multiscanner study

A second, smaller study was run to determine the reproducibility of T1 maps across different clinical scanners. Two healthy volunteers (38.5 ± 3.5 years, both females) were scanned with a T1‐weighted MPRAGE sequence on six various 3 T MRI scanners: 2 Philips Achieva, a Philips Ingenia, 2 GE Discovery MR750w GEM, and a GE Signa Architect. The scan parameters for each are listed below in Table 2. Note the large range or TI values (which determines T1‐weighting) vary from 450 to 1010 ms in this study. Images were converted into T1 maps via T1‐REQUIRE. No T1 mapping reference sequence was used since this was purely an exploratory study to determine the reproducibility of T1‐REQUIRE across various scanners types and manufacturers.

**TABLE 2:** Scan parameters for multiscanner experiment

	GE Discovery #1	GE Discovery #2	GE Signa Architect	Philips Achieva #1	Philips Achieva #2	Philips Ingenia
Orientation	Axial	Axial	Axial	Axial	Axial	Axial
#Slices	182	174	174	160	180	167
Slice thickness (mm)	1	1	1	1	1	1
FOV (mm)	256/256	256/256	250/256	250/199	250/225	240/240
Voxel size (mm)	1 × 1 × 1	1 × 1 × 1	1 × 1 × 1	1 × 1 × 1	1 × 1 × 1	1 × 1 × 1
*TR* (ms)	8.5	8.6	7.9	7.9	6.9	8.1
*TE* (ms)	3.2	3.2	3.1	3.5	3.5	3.7
*TI* (ms)	450	450	450	827.7	810	1010
# Channels	16	24	16	32	16	8

Abbreviations: GE, General Electric; mm, millimeters; ms, milliseconds.

### 2.8 Data analyses

All data analyses were completed successfully in MATLAB without software failures or user input. For the first study, correlation plots were constructed, and linear regression was performed to determine the correlation slopes and correlation coefficients (both Pearson's correlation coefficient and Lin's concordance correlation coefficient) between the reference standard Look‐Locker mapping sequence and the T1‐REQUIRE method with both T1‐weighted sequences. In addition, both T1‐weighted sequences were co‐registered with the Look‐Locker reference images using SPMv12 for a more accurate comparison before conversion to T1 maps. The brain was segmented via SPMv12 before and 4 × 4 × 1 voxel smoothing kernel was convolved with the T1‐REQUIRE maps and Look‐Locker map to reduce noise prior to comparison. For the second study, no reference T1 mapping image was acquired. Instead, the T1‐REQUIRE algorithm converted the MPRAGE images into T1 maps for comparison with each other. The T1‐weighted images were registered with each other for each individual subject before T1‐REQUIRE was run. After conversion to T1 maps, a 4 × 4 × 1 smoothing kernel was convolved with the maps and T1‐weighted images to reduce noise prior to comparison. Linear regression was run between maps converted from each sequence and the smoothed T1‐weighted images, and the results were plotted to compare with the line of unity. In addition, a cumulative distribution function (CDF) was plotted for each T1‐weighted image and T1 map to examine the degree of congruence of the data.

## 3 RESULTS

### 3.1 T1‐REQUIRE versus Look‐Locker

**FIGURE 4:**
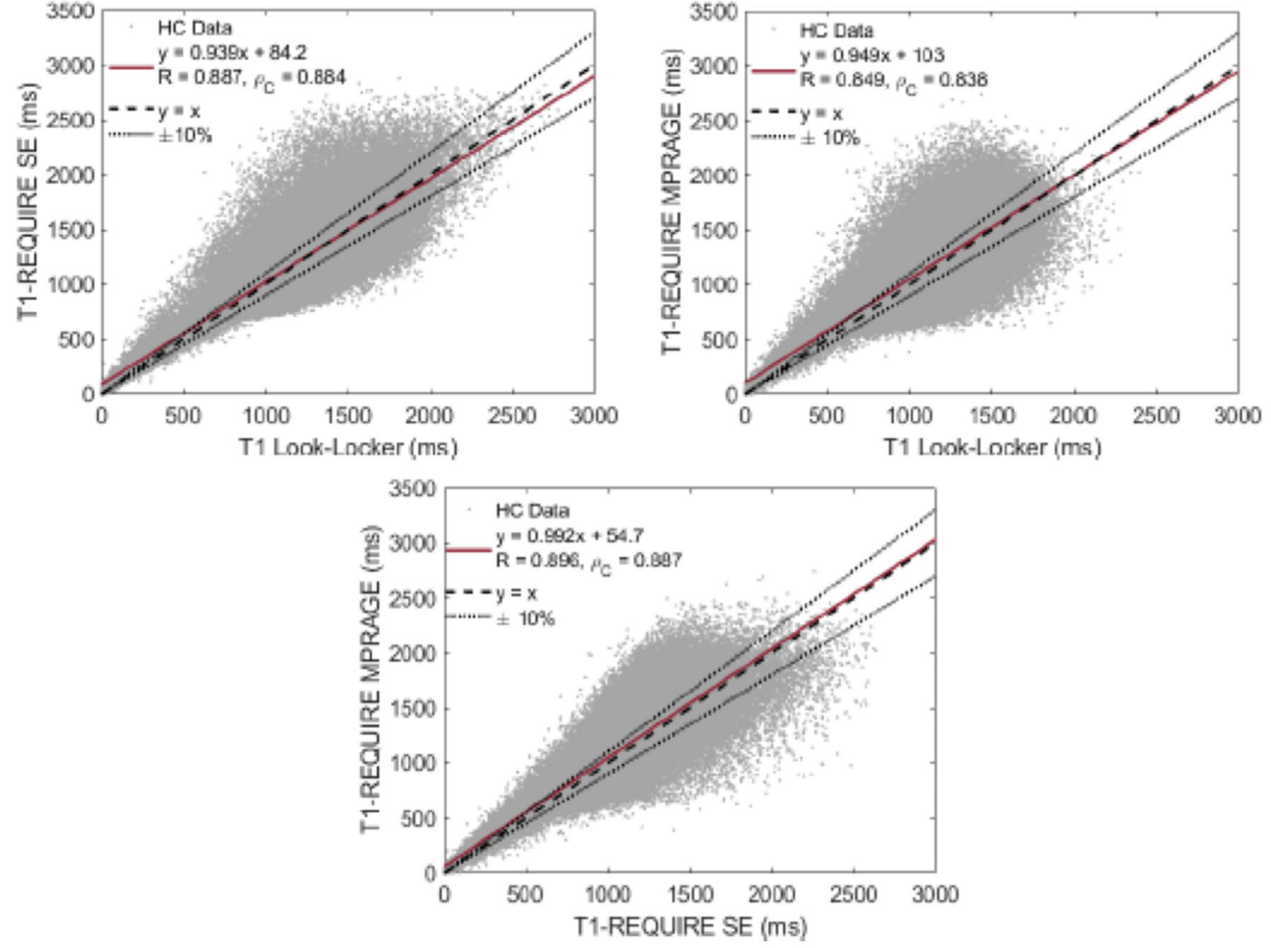
Correlation plot between corrected Look‐Locker T1 relaxation times and those produced by T1‐REQUIRE on spin‐echo images (top left), corrected Look‐Locker T1 relaxation times and those produced by T1‐REQUIRE on MPRAGE images (top right), and T1 relaxation times from T1‐REQUIRE on spin‐echo images and those produced by T1‐REQUIRE on MPRAGE images (bottom), all in healthy controls. Black dotted lines indicate 10% variation from the black dashed line of unity. Where the solid red fit line is within the ±10% lines in the top left and top right images indicate the range of T1 values where T1‐REQUIRE is acceptable.

The resulting comparison of T1 relaxation times from the 10 healthy controls using the Look‐Locker reference standard, T1‐REQUIRE with the spin‐echo MRIs, and T1‐REQUIRE with the MPRAGE MRIs are shown in Figure 4. For each data set, the T1‐REQUIRE method was completed in less than a minute on a spin‐echo image, including the conversion of files from DICOM to NIFTI formatting, and the conversion of multiple TI Look‐Locker images to T1 maps was completed in an average 106 min on the same machine at the same resolution, a more than ×100 increase in computational efficiency. For T1‐REQUIRE on the MPRAGE images, the average computation time was around 7 min for a full estimated 3D T1‐map, a ×15 increase in computational efficiency even with a superior resolution over the Look‐Locker images. When T1‐REQUIRE was run on the registered MPRAGE images, the average computation time was just over a minute, again more than a ×100 increase in computation time.

When comparing the T1 relaxation times from the T1‐REQUIRE on spin‐echo images with those from the reference standard Look‐Locker, we found a correlation slope of 0.939, intercept of 84.2 ms, Pearson's correlation coefficient of 0.887, and Lin's concordance correlation coefficient of 0.884. This small difference between the Pearson's and Lin's correlation coefficients indicate a very slight bias in our method, but nothing significant. Using the linear regression fit, we can calculate an effective range for T1‐REQUIRE on T1‐weighted spin‐echo images as 523–3000 ms, the limit of our analysis.

**FIGURE 5:**
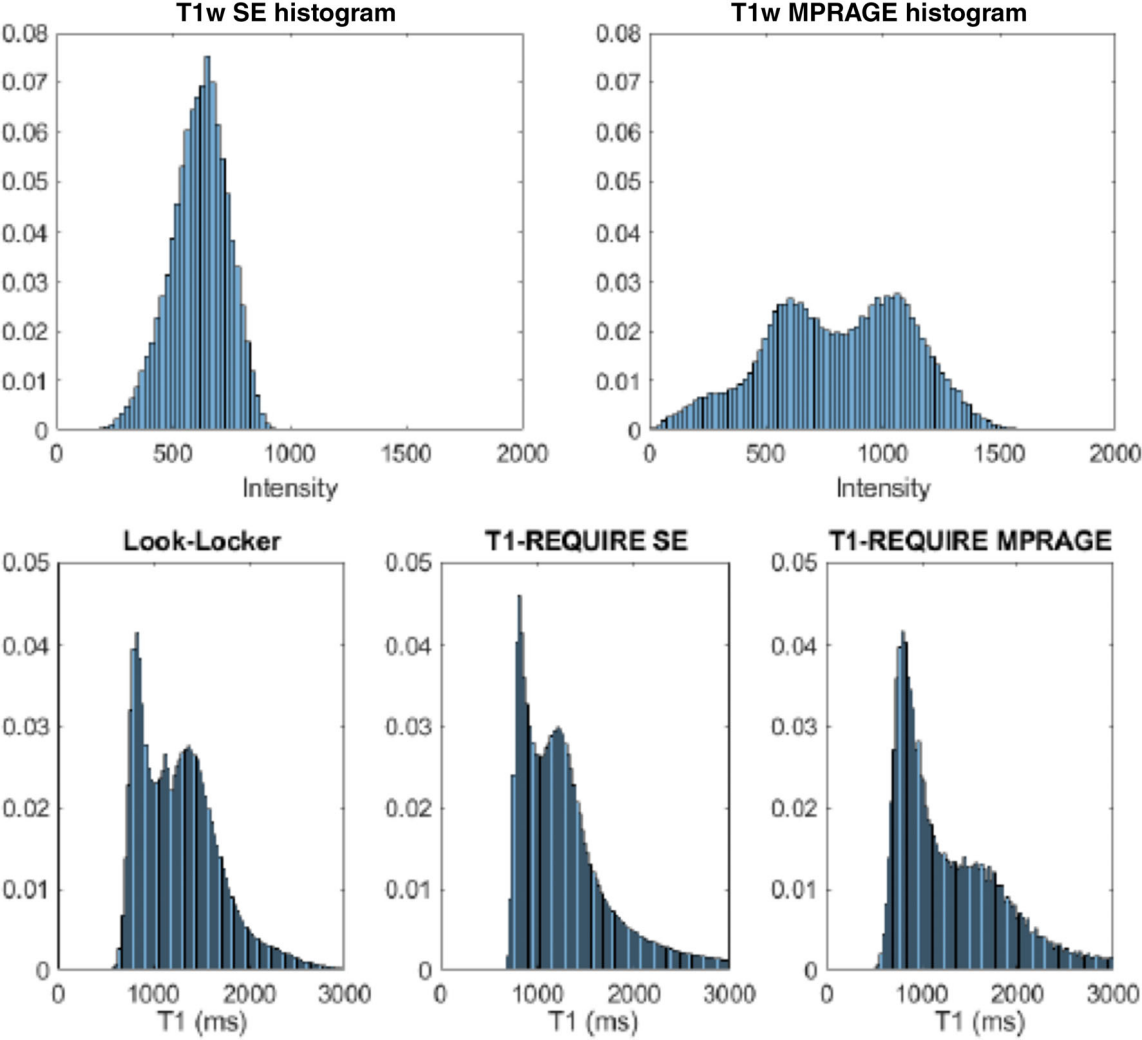
Histograms of T1‐weighted spin‐echo images (top left) and T1‐weighted MPRAGE images (top right), and histograms of T1 values from reference Look‐Locker (bottom left), T1‐ REQUIRE with spin‐echo images (bottom middle), and T1‐REQUIRE with MPRAGE images (bottom right) from the same 10 healthy control brains. The bottom histograms show the same general shape with two distinct peaks, unlike with the histograms on the top.

**FIGURE 6:**
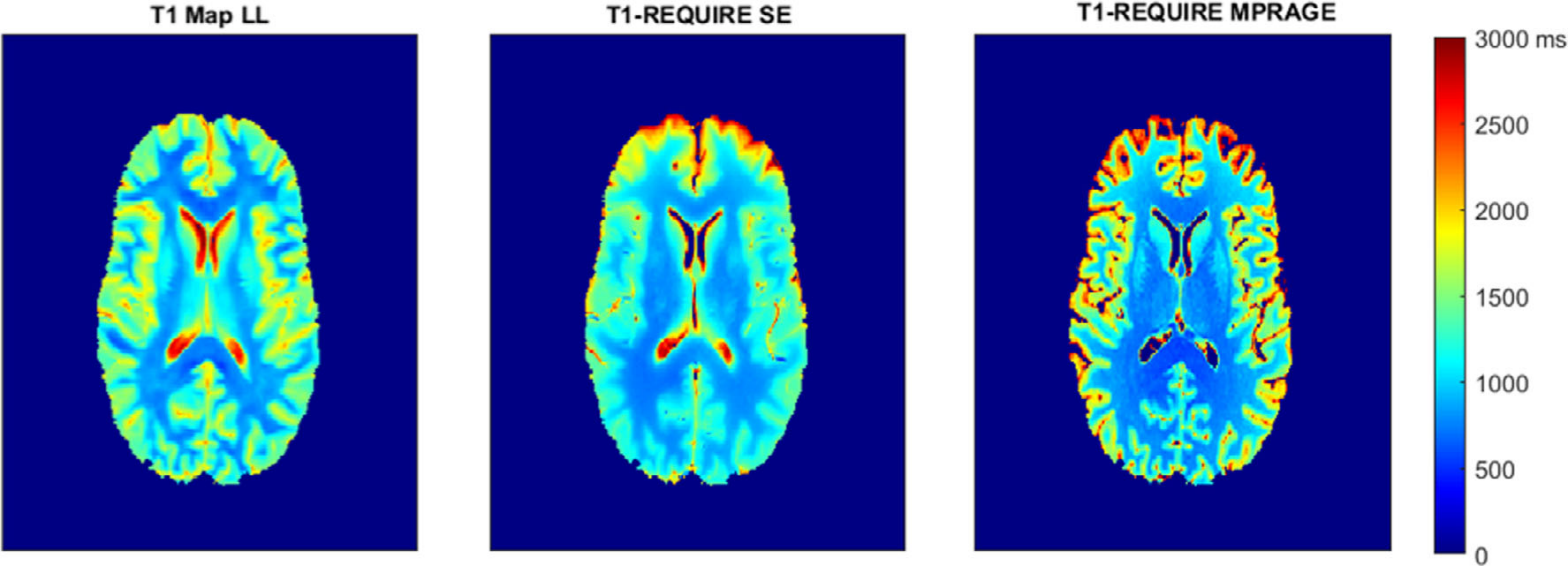
Example T1 maps generated by the reference standard Look‐Locker (left), T1‐REQUIRE on spin‐echo images (center), and T1‐REQUIRE on MPRAGE images (right)

When comparing the T1 relaxation times from the T1‐REQUIRE on MPRAGE images with those from the reference standard Look‐Locker, linear regression produced a correlation slope of 0.949, intercept of 416 ms, Pearson's correlation coefficient of 0.849, and Lin's concordance correlation coefficient of 0.838. Again, there is a slight difference between the two correlation coefficients, but nothing concerning. Using the fit, we can calculate an effective range for T1‐REQUIRE on T1‐weighted MPRAGE images as 682–3000 ms. To be effective as a data congruence algorithm and hence optimal for data curation, it is important that T1‐REQUIRE achieve the same or very similar results regardless of what sort of T1‐weighted sequence is run. In that regard, we can do a similar analysis on T1‐REQUIRE using both the spin‐echo and MPRAGE images. Linear regression produces a correlation slope of 0.992, intercept of 54.7, Pearson's correlation coefficient of 0.896, and Lin's concordance correlation coefficient of 0.887, showing good agreement between the two with very little bias. In addition to correlation plots, the histograms of the culmination of the 10 healthy controls are shown in Figure 5A,B. Figure 5A shows the histograms of the weighted images, while Figure 5B shows those of the estimated and reference T1 maps. Although the T1 maps differ slightly, especially comparing the T1‐REQUIRE from the spin‐echo image (center) with the reference T1 map (left), the same general shape can be seen, showing improved data congruence over the T1‐weighted images. Representative slices of the three T1 maps are shown in Figure 6.

### 3.2 T1‐REQUIRE with and without RAVEL harmonization

**FIGURE 7:**
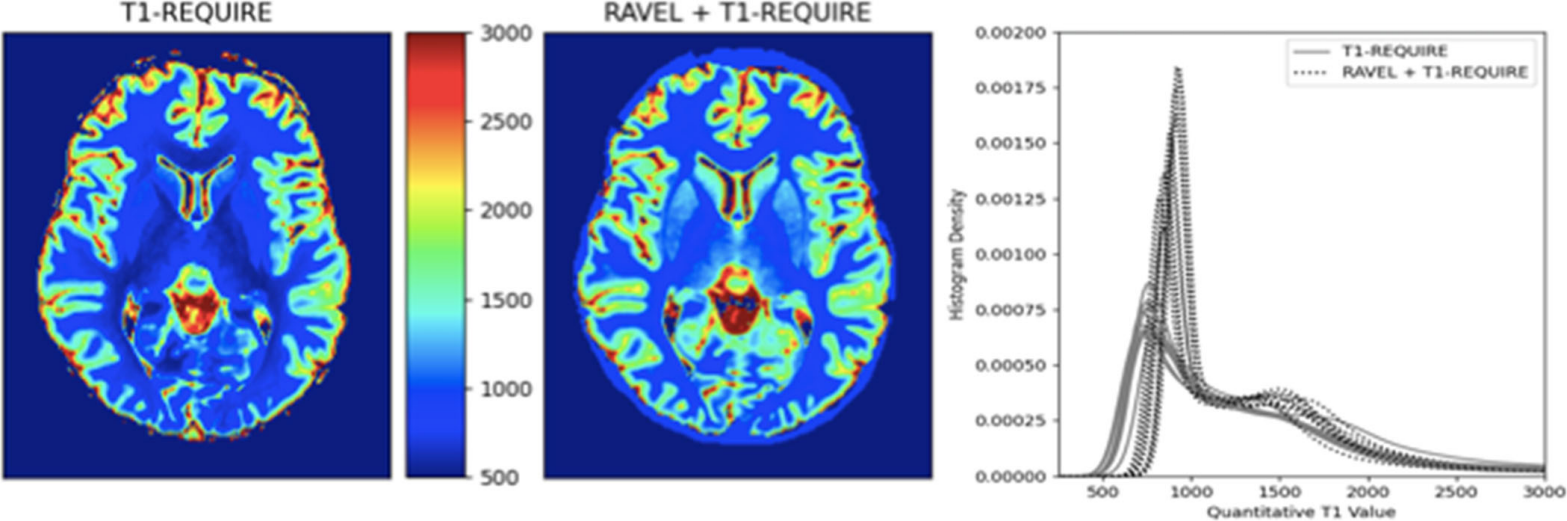
Comparisons of T1 relaxation maps (left) without intensity normalization and (center) post RAVEL intensity harmonization and (right) associated histogram density plots

From visual inspection, RAVEL intensity normalization did not significantly influence the quality of T1 maps. Also, the density distribution of voxels in the T1 maps were distributed over a more narrow range after RAVEL harmonization (Figure 7).

### 3.3 Multiscanner study

**FIGURE 8:**
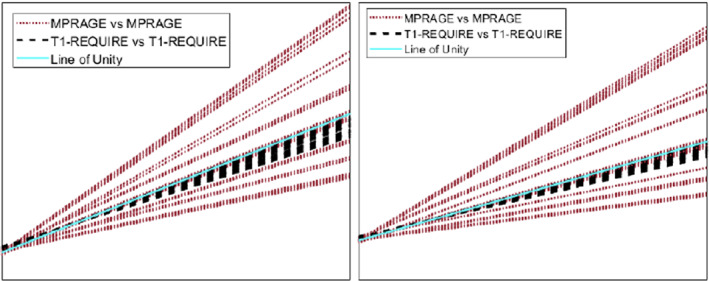
Results from linear regression analysis comparing MPRAGE versus MPRAGE T1‐weighted images (red dotted line) and corresponding T1‐REQUIRE versus T1‐REQUIRE (black dashed line) from Subject 1 (left) and Subject 2 (right) taken on different scanners, along with the line of unity (cyan). The black dashed lines cluster more closely around the line of unity, indicating much improved harmonization compared to the red dotted lines

**FIGURE 9:**
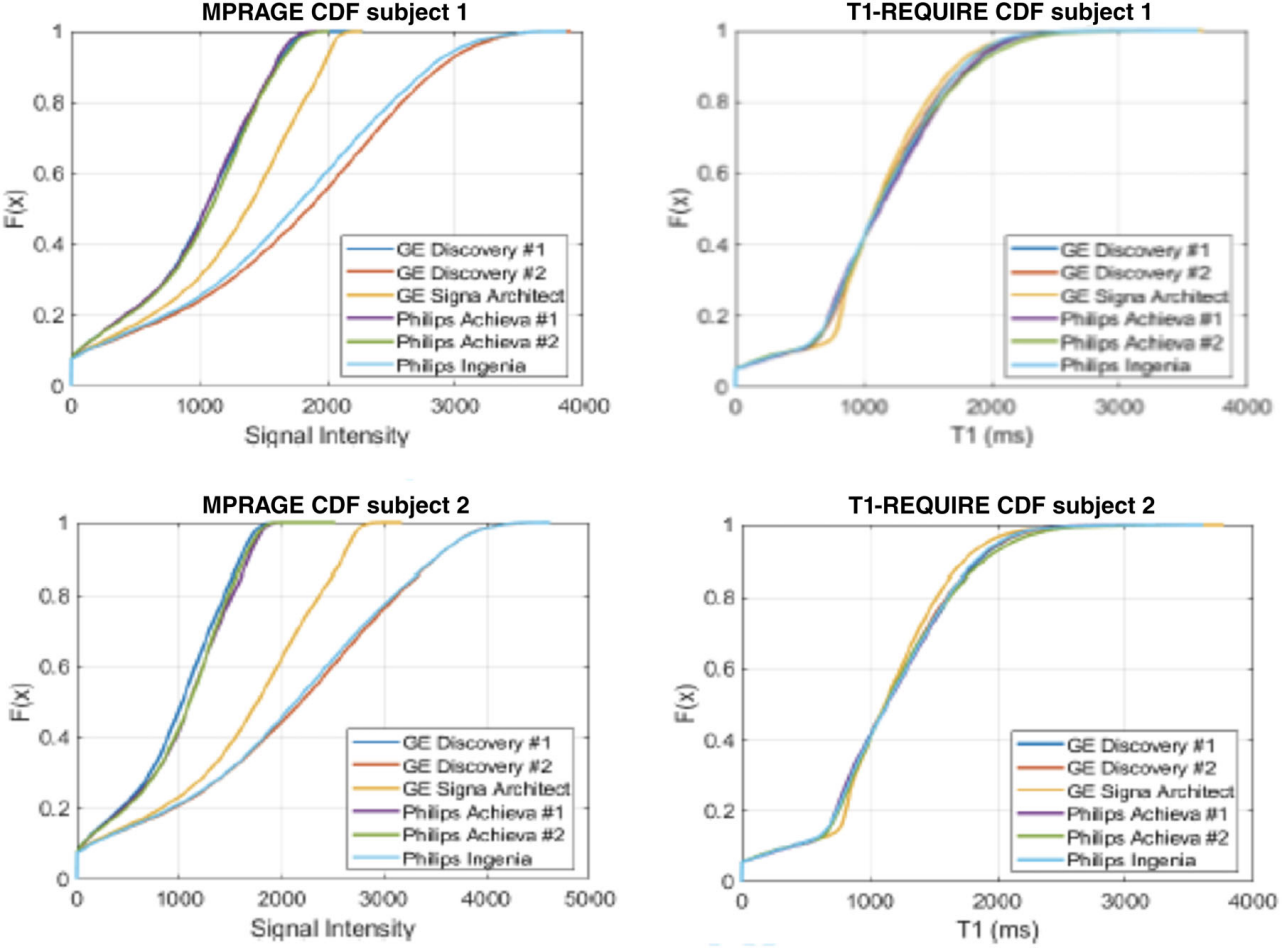
Comparison of CDFs from Subject 1 (top) and Subject 2 (bottom) for both the MPRAGE images (left) and T1‐REQUIRE maps (right) from six different scanners. For both subjects, there was clear improvement in the normalization of the CDFs after applying T1‐REQUIRE.

Figure 8 shows the results of the linear regression between either the original MPRAGE images (red dotted line) and T1‐REQUIRE (black dashed line), along with the line of unity (cyan filled line) for both Subject 1 (left) and Subject 2 (right). Visual inspection shows that the spread of comparisons between T1 maps generated via T1‐REQUIRE is much more consistent with each other and with the line of unity, providing evidence that T1‐REQUIRE can be adapted to images across scanners. This result is consistent with the CDFs shown in Figure 9, where the MPRAGE images have drastically varying CDFs that are much more uniform after T1‐REQUIRE is completed, a conformity built in the methodology of T1‐REQUIRE implementation.

## 4 DISCUSSION

In this study, it was shown that the T1‐REQUIRE algorithm developed to take previously acquired, T1‐weighted images and convert them into T1 maps resulted in a good estimation of the reference standard between 500 and 3000 ms for a T1‐weighted spin‐echo and 680–3000 ms for a 3D MPRAGE. This is an acceptable range of T1 values to operate within for structural neuroimaging, as both WM and GM fall within that range and any pathology to those structures would likely fall within it as well. In addition, T1‐REQUIRE showed good agreement across different T1‐weighted sequences and across scanner type and parameter differences, making it a potentially useful tool for analyzing T1 relaxometry alterations in various disease states independent of scanner utilization.

From the histograms in Figure 5, we can see a marked improvement in the normalization of the two weighted scans, a spin‐echo and MPRAGE, into estimated T1 maps. The images show relatively good visual agreement between the three methods, even in the deep gray matter of the putamen and caudate nucleus. In addition, the T1 map generated by T1‐REQUIRE on the T1‐weighted spin‐echo image has a histogram that looks remarkably like that of the reference standard Look‐Locker. However, the map generated by the MPRAGE has a noticeably drawn out gray matter peak. More work is being completed to confirm the source of this phenomenon. Some preliminary thoughts are that it is due to partial volume effects between the gray matter and CSF. Because the MPRAGE has a slightly lower resolution in one dimension, there could be additional blurring from the gray matter and CSF that smooths out the normal gray matter peak on the histogram. That being said, T1‐REQUIRE did produce a histogram from the MPRAGE image that matches the general shape of the other two histograms. In addition, the second study showed remarkable conformity of the CDFs from the same subject scanned across various scanners and with different scan parameters. The initial MPRAGE images resulted in drastically different histograms and CDFs, as expected when scan parameters are varied, and different filters and reconstruction techniques are used. However, it seems that T1‐REQUIRE was able to overcome most of these challenges and produce an image that is reproducible across scanners. Furthermore, we tested the inherent ability of T1‐REQUIRE in mitigating the variability in T1 values across subjects and compared this algorithm to RAVEL intensity normalization followed by T1‐REQUIRE. Both of these procedures produce very similar results as confirmed by visual inspection (Figure 7). It appears that there is a slightly greater variability seen in the T1 relaxation density plots after T1‐REQUIRE, which is mitigated when RAVEL is performed first (narrow histogram peaks). It remains to be determined in the future ongoing studies whether this variability is of biological relevance as determined by better prediction with clinical data. Overall, our results provide evidence that T1‐REQUIRE is easily acquired and data is inherently aligned with each other across subjects and scanners and hence these features may be very useful for machine learning pipelines.

Of note, the goal of T1‐REQUIRE is to retrospectively quantify tissue T1 relaxometry for potentially examining intrinsic pathology and not for the purpose of quantification of brain volumes for atrophy analysis. Our approach is a departure from prior techniques such as RAVEL, which was shown to improve upon the so‐call White Stripe statistical normalization algorithm of signal intensities by selecting target tissues and imposing piecewise linear regression to alter the relative signal intensities of healthy tissue (GM, WM).^3^ Although an improvement over White Stripe normalization, RAVEL does not provide an absolute scale to image, which is the goal of T1‐REQUIRE. Rather than assuming a linear dependence, we explicitly incorporate the physics non‐linearity and remove the arbitrariness resulting from tuning receiver gains that is inherent to the MRI signal. In doing so, we provide an absolute scale (T1 value) that is indicative of the degree of tissue change, not simply the presence or absence of pathology. Direct quantification of tissue properties allows more subtle evaluation of disease beyond presence/absence of disease that volumetric analysis provides. For example, the mixing of two T1 species in a voxel (myelinated neurons and demyelinated neurons for example) is given by:

$$T_{1}^{\text{VOXEL}}=\left(\frac{T_{1}^{a}\times T_{1}^{b}}{\beta T_{1}^{a}+\propto T_{1}^{b}}\right)$$

Providing a true conversion of signal intensity to T1 will facilitate quantifying the *degree* of pathologic change (determination of alpha, beta per voxel), not just the *presence* of pathologic change. Future work will apply this normalization to quantify tissue pathology to various diseases, further validating the usefulness of T1_REQUIRE.

REQUIRE algorithm may appear somewhat circular in definition, that is, the values for the average pixel intensities for each tissue types are set by historical literature values. However, the purpose of the first half of this study was to discern if this was an appropriate way to estimate T1. Our results mitigate this concern since it was shown that T1‐REQUIRE is a good approximation of T1 as compared with the reference standard T1 mapping method (which is NOT dependent on prior knowledge). This suggests that brain tissue in most people have approximately the same T1 value as the historical references, which we have shown is an appropriate assumption to make at the 10% level of accuracy. Conformity of MRI data is an important aspect of machine learning that is often overlooked or understated. Without it, any results may be skewed by the variability in scan‐ or scanner‐specific properties. Relaxometry could have the dual ability to provide data conformity along with providing interesting, tissue‐specific information not previously seen. However, the downsides of standard relaxometry measurements often outweigh the benefit for clinicians, resulting in a lack of relaxometry datasets that are suitable for analysis via machine learning. By providing estimates of T1 using T1‐weighted images, previously acquired weighted images can be converted efficiently to estimate T1 maps for big data studies.

Historically, MR relaxometry can be fit in a variety of ways, including an IR sequence with multiple TIs and a spin‐echo with multiple repetition times. Such methods have the advantage of accuracy but require long scan times prohibiting use in clinical studies. Other sequences have been developed to reduce scan times, such as the Look‐Locker method and MR Fingerprinting (MRF). These can be implemented as a more‐time‐efficient method of acquiring quantitative MR parameters like T1.^20^ But, as time efficient as they can be, both are still an additional scan that clinicians do not often order or use for their clinical analysis of disease. In addition, MRF was only introduced within the last 5 years, meaning a large portion of information even in current datasets would not have been able to run a MRF sequence. So, while MRF is a useful tool, it is a prospective tool with no applications retrospectively and it is not frequently utilized by clinicians. T1‐REQUIRE can be used retrospectively with no additional sequences required, making it a useful addition to workflow for estimating T1. The historical T1, T2, and proton density values, found in the literature, present a challenge, as T1 values vary across the same tissue type depending on location (i.e., parietal GM having a lower T1 than frontal gray matter: 1276–1322 ms, respectively).^13^ In addition, intra‐ and interscanner variability may result in additional error, as studies have found a 1% intrascanner variability, 1% intravendor/interscanner variability, and a 8%–10% intervendor variability across the whole brain due to factors like B_0_ field differences, B_1_ map variations, shimming procedure, and so on.^13^ Future work is ongoing to validate T1‐REQUIRE in various disease states and examine whether biological variance can still be detected despite some of the fixed sources of variability inherent in analyzing brain images.

Some assumptions are made in the T1‐REQUIRE algorithm. For the T2 correction, this may not be a totally accurate assumption, as T2 varies along with T1 in diseased tissue.

However, error propagation analysis (derived from Equation (1) showing how *S* varies with changes in T1 and T2 before equating them) shows that the true T2 value of a voxel of average gray matter having an uncertainty of 50% would result in only a 5.6% uncertainty in T1, and the same in a voxel of average white matter would result in an uncertainty of 8.7% (Equations ((5a), (5b), (5c))). Because of this, we argue that the estimation of T2 step for T1‐REQUIRE of a spin‐echo remains appropriate except in the most extreme cases of changes in T2. This is also exemplified in Figure 6, where the putamen and caudate nuclei visually appear to have T1 values relatively consistent with the literature even with the variation of T2 in deep gray matter.

(5a) δSS=TR⋅δT1T12eTRT1−1

(5b) $$\frac{\mathrm{\delta S}}{S}=\frac{\mathrm{TE}\cdot \mathrm{\delta T}2}{T2^{2}}$$

(5c) δT1T1=TE⋅δT2⋅T1eTRT1−1TR⋅T22

Factors such as coil sensitivity and flip angle variability were also ignored, along with the previous assumptions about T2 and proton density distributions. While these may not be appropriate assumptions, a large part of the application of T1‐REQUIRE lies in retrospective studies where more information may not be available for use. The structure of the studies presented was to determine whether using information gained from either the literature or the T1‐weighted image itself would allow for a reasonable estimation of a T1 map that could be reproduced over multiple scanners.

This pilot study shows the potential viability of T1‐REQUIRE for retrospective estimation of relaxation times from weighted MR data as both an efficient estimation of T1 and a data conformity and curation technique for machine learning applications. More work needs to be done to expand the REQUIRE algorithm to other T1‐ and T2‐weighted imaging sequences as well as validation of its potential in machine learning pipelines.

## Data Availability

The data that support the findings of this study are available from the corresponding author upon reasonable request.
